# Can Leucine Supplementation Improve Frailty Index Scores?

**DOI:** 10.3390/geriatrics8050102

**Published:** 2023-10-12

**Authors:** Cristina Buigues, Olga Theou, Rosa Fonfría-Vivas, Francisco M. Martínez-Arnau, Kenneth Rockwood, Omar Cauli

**Affiliations:** 1 Frailty and Cognitive Impairment Research Group (FROG), University of Valencia, 46010 Valencia, Spain; cristina.buigues@uv.es (C.B.); rosa.fonfria@uv.es (R.F.-V.); francisco.m.martinez@uv.es (F.M.M.-A.); 2 Department of Nursing, University of Valencia, 46010 Valencia, Spain; 3 Department of Medicine, Dalhousie University, 5955 Veterans’ Memorial Lane, Halifax, NS B3H 2E1, Canada; olga.theou@dal.ca (O.T.); kenneth.rockwood@dal.ca (K.R.); 4 School of Physiotherapy, Faculty of Health, Dalhousie University, Halifax, NS B3H 4R2, Canada; 5 Department of Physiotherapy, University of Valencia, 46010 Valencia, Spain

**Keywords:** frailty index, skeletal muscle, muscular strength, nursing home, randomized clinical trial

## Abstract

Sarcopenia and frailty are important conditions that become increasingly prevalent with age. There is partial overlap between the two conditions, especially in terms of the physical aspects of the frailty phenotype: low grip strength, gait speed, and muscle mass. This study examined whether administration of the essential branched-chain amino acid leucine, besides improving sarcopenia, may reduce frailty assessed by frailty index (FI) in older institutionalized people living in nursing homes. We conducted a secondary analysis of a placebo-controlled, randomized, double-blind design study (ClinicalTrials.gov NCT03831399). The study included fifty males and females aged 65 and over who were living in nursing homes and did not have dementia. The participants were randomized to a parallel group intervention of 13 weeks’ duration, with a daily intake of leucine (6 g/day) or placebo (lactose, 6 g/day). The outcome of this study was to evaluate whether there was a change in the level of a 95 item FI compared to the baseline and to compare the effect of the leucine group versus the placebo group. A significant inverse correlation was found between FI and performance of the activities of daily life, cognitive function, gait and balance, muscle function parameters, and nutritional status (*p* < 0.001 in all cases). There were no statistically significant differences in FI levels at baseline (placebo group FI 0.27 ± 0.08 and leucine group FI 0.27 ± 0.10) and at the 13 week follow-up (placebo group FI 0.28 ± 0.10 and leucine group FI 0.28 ± 0.09). There were also no significant differences between the leucine and placebo groups in the mean FI difference between baseline and follow-up (*p* = 0.316, Cohen’s d: 0.04). This pilot study showed that a nutritional supplementation with leucine did not significantly modify the frailty index in older nursing home residents.

## 1. Introduction

Functional decline in the elderly is associated with sarcopenia and frailty, both of which are becoming increasingly prevalent with progressive ageing of the population, and they are very prevalent in institutionalized elderly people who accumulate several deficits and suffer from several associated comorbidities. Although these two entities partially overlap as they have some similar pathophysiological causes, they are distinct in several respects, with frailty being a more complex syndrome with multiple bio-psycho-social mechanisms involved. Sarcopenia is defined as a loss of muscle mass, strength, and function [1], whereas frailty is a state of vulnerability arising from the impairment of multiple physiological systems and is associated with aging, but its prevalence varies widely among older people [2]. Frailty can be measured using several tools, including the frailty index [3] and the frailty phenotype [4], among the most widely used in the literature. Many physiological systems have been studied for their relationship to physical frailty [5,6,7], but skeletal muscle loss has been one of the commonly observed features [8,9]. If the physical frailty phenotype is considered, there is an overlap with sarcopenia, being characterized by low grip strength and decreased gait speed [10,11,12]. In addition, sarcopenia may be a risk factor for frailty defined as a physical phenotype [13,14,15,16]. Both sarcopenia and frailty share common pathogenic mechanisms [17] and some physical diagnostic items (low grip strength and gait speed). Both syndromes are considered to be the main causes of functional impairment in older people, leading to disability, falls, poor quality of life, institutionalization, and mortality. The evidence collected so far has mainly focused on the associations of frailty or sarcopenia taken separately on the adverse health outcomes cited above.

A recent longitudinal study has shown in a community-dwelling population that only a small proportion of sarcopenic individuals are frail, ranging from 8.2% to 15.7% depending on the diagnostic criteria used for sarcopenia, whereas about one-third of frail individuals do not have sarcopenia [18]. This finding suggests that sarcopenia and frailty are related entities but are not the same condition and that their association may possibly respond to the existence of different clinical forms of frailty (sarcopenic and non-sarcopenic) with different pathophysiological backgrounds and different risks of adverse outcomes [19].

There is good scientific evidence that nutritional interventions based on protein and branched-chain amino acid (BCAA) (valine, leucine, and isoleucine) are effective for a proper maintenance of muscle mass and function and they are a crucial element of the nutritional approach in sarcopenia. In addition, BCAAs have anabolic effects that are associated with an increase in protein synthesis and a reduction in the rate of protein degradation [20,21,22]. Supplementation with BCAAs or proteins rich in these amino acids had positive effects on sarcopenia [23,24,25]. A recent randomized placebo-controlled clinical trial (RCT) demonstrated that supplementation with the BCAA leucine for 13 weeks improved some sarcopenia criteria in institutionalized elderly people, as was an improvement in functional performance measured by gait speed and improvement in lean mass index [26]. As part of the Seniors-ENRICA study, the effects of dietary leucine on frailty syndrome were studied, showing that participants in the highest tertile of leucine intake had a lower risk of incident frailty, as measured by Fried’s phenotype criteria [27].

Studies of nutritional intervention in older people with amino acids and/or protein have mainly been directed at the assessment of sarcopenia, but their efficacy in frailty syndrome has been studied much less frequently. Most published studies on this topic suggest that modification of nutritional quality, either through supplementation or improved dietary intake, could improve strength, gait speed, and nutritional status in most frail or pre-frail older adults. However, there is a lack of scientific evidence to recommend these interventions to at least partially reverse frailty as a reversible syndrome.

No studies have been published on the effects of leucine supplementation on frailty index, defined as the proportion of deficits present in an individual out of the total number of age-related health variables considered. A frailty index can be created in most secondary data sources related to health by utilizing health deficits that are routinely collected in health assessments. These deficits include diseases, signs and symptoms, laboratory abnormalities, cognitive impairments, and disabilities in activities of daily living [3,28].

Early identification and management of frailty is relevant to achieve the goal of healthy aging. The frailty index (FI) may be a more suitable instrument to assess change in frailty after intervention because it has a continuous scoring system and includes more items from various domains than the frailty phenotype [29].

The aim of this secondary analysis was to analyze the data from the previous registered RCT to assess the effect of leucine supplementation on the level of frailty as measured by the frailty index (FI).

## 2. Materials and Methods

We conducted an exploratory secondary analysis of a placebo-controlled, randomized, double-blind design study on leucine supplementation. The methodology of the primary study has previously been described in detail [26]. The methodology and data evaluation and presentation was based on a secondary analysis of a RCT aimed to evaluate the effect of nutritional supplementation in frailty index by Theou and co-workers [27]. Briefly, older (>65 years) participants with no dementia who were living in three nursing homes in Valencia, Spain, and able to walk 6 m were recruited. The participants were randomly assigned to a parallel group intervention of 13 weeks’ duration with a daily intake of L-leucine or the placebo (lactose), in powder form. The nurses, or the participants themselves under the supervision of a nurse, dissolved two tablespoons of L-leucine (6 g/day) or placebo (lactose, 6 g/day) in a glass of water or juice. Either the L-leucine or placebo was administered after breakfast (between 9 and 10 a.m.) and in the afternoon at 4–5 p.m. (approximately 3 g).

Sarcopenia was measured according to the guidelines of the European Working Group on Sarcopenia in Older People (EWGSOP) [1], as published in the original trial in 2020 [26], and can be assessed by means of indirect measures of muscle function and muscle mass, such as low walking speed (0.8 m/s walking 4.6 m), handgrip strength assessed by dynamometry (for men 30 kg/m^2^ and for women 20 kg/m^2^), and the loss of lean mass calculated using the equation formulated by Janssen [17].

Muscle mass was determined using the formula of Janssen et al. [17]: muscle mass (kg) = [(height^2^/R 0.401) + (3.825 sex) + (0.701 age) + 5102, where height is expressed in cm, R in ohms, and age in years, and female sex has a value of zero and males a value of one. The muscle mass index (MMI) is established as the muscle mass a person has, corrected by body surface area (muscle mass/height^2^). Muscular strength was measured in the dominant hand with a handgrip dynamometer (Saehan Smedley Hand Dynamometer^®^ Yangdeok-Dong, Masan 630-728, Korea), and the test was always replicated three times during a 5 min period, with the mean value of the trials being recorded.

Frailty levels were based on the deficit accumulation approach and were measured using an FI [30]. The FI was constructed following standard procedures [28]. Each item included in the FI was age related, available at both baseline and follow-up, and when combined covered several organ systems. We screened all variables collected as part of this RCT and constructed the FI using 95 variables; the included items had missing data for 5 or less people at each time point (baseline and follow up). We included items from the Barthel Index (10 items), Tinetti Balance and Gait Evaluation (14 items), Mini Nutritional Assessment (13 items), Mini-Mental State Examination (13 items), anthropometrics (1 item), comorbidities (11 items), blood tests (21 items), and others (12 items). Each item included was mapped on a 0 to 1 interval, with a value of 0 when the deficit was absent and 1 when the deficit was fully expressed. Each patient’s FI score was calculated by dividing the number of deficits by the number of total variables measured for that participant (e.g., 95 variables if a participant was missing one item). The FI score is thereby continuous (0–1). We calculated an FI score for each participant at both baseline and follow-up only if fewer than 16% of variables were missing (at least 80 of 95 items available at each time point) [31]. We also classified people in frailty groups: non-frail (0–0.1), very mildly frail (0.1–0.2), mildly frail (0.2–0.3), moderately frail (0.3–0.4), and severely frail (>0.4); the first two frailty groups were combined, as were the last two frailty groups, as only one person was included in the non-frail group and three people in the severely frail group.

Sample size estimation was calculated accepting an alpha risk of 0.05 and a beta risk of 0.2 in a two-sided test; 18 subjects were necessary in first group and 18.0 in the second to recognize as statistically significant a difference greater than or equal to 0.3 units. The common standard deviation of FI was assumed to be 0.3 and the correlation coefficient between the initial and final measurement as 0.5. We anticipated a drop-out rate of 10%.

The study was conducted according to the guidelines set by the Declaration of Helsinki, and all procedures involving human participants were approved by the Ethics Committee of the University of Valencia (Valencia, Spain—H1424156718665). Written informed consent was obtained from all participants.

### Statistical Analyses

We used descriptive statistics with mean and standard deviation for continuous variables and frequency for categorical variables. The differences in quantitative variables between two independent groups (leucine versus placebo group) were analyzed with the Mann–Whitney U-test. Spearman’s non-parametric test was used to analyze the correlation between the quantitative variables depending on their distribution. We compared frailty scores at baseline and at 12 week follow-up using a mixed-design variance analysis; the treatment group (intervention or placebo) was the between-subjects factor, and time was the within-subjects factor. To quantify the clinical detectability of treatment effects, we calculated Cohen’s d and the standardized response mean (mean change divided by the standard deviation of the change scores). The analyses were conducted using SPSS (version 24, SPSS Inc., Chicago, IL, USA). The level of statistical significance was set at a *p* value of 0.05. To quantify the clinical detectability of treatment effects, we calculated Cohen’s d and the standardized response mean (mean change divided by the standard deviation of the change scores).

## 3. Results

It was possible to calculate the FI of 41 participants who had sufficient data to calculate it in both measurements (before and after leucine or placebo administration); one participant in the placebo group was excluded because he was missing 34 elements to calculate the FI at baseline (before placebo administration). The mean age of the participants was 78.2 ± 9.1 years, and most of them were women (n = 27, 66%). The participants lived in three different nursing homes. The mean value of the FI at baseline was 0.27 ± 0.09 (range 0.08–0.49, median 0.30), and 9 (22%) participants were classified as non-fragile or very mildly fragile (FI ≤ 0.2), 18 (43.9%) as mildly fragile (FI 0.2–0.3), and 14 (34.1%) as moderately/severely fragile (FI > 0.3). There were not significant differences in FI among the participants from the three nursing homes) (nursing home A (n = 14): 0.307 ± SE 0.0265; nursing home B (n = 16) 0.253 ± SE 0.2319; nursing home C (n = 11) 0.2514 ± SE 0.245 *p* = 0.272; Kruskal–Wallis test).

The FI had a normal distribution at baseline and was not significantly related to the age of the participants analyzed as a continuous variable (r = 0.303; *p* = 0.054). Dichotomizing the age, we also observed no significant difference between FI scores at baseline among participants older than 80 years (N = 22; 0. 29 ± 0. 07) compared to those aged 80 years or younger (N = 19; 0.24 ± 0.10; *p* = 0.072). There was no significant difference in FI at baseline between men and women (in men 0.27 ± 0.10 and in women 0.26 ± 0.08; *p* = 0.425). There were no statistically significant differences between the intervention and placebo groups in terms of age (*p* = 0.989), gender distribution (*p* = 0.520), FI levels (*p* = 0.438), and FI group distribution (*p* = 0.898) at baseline. The two experimental groups (leucine and placebo) were not significantly different at baseline (Table 1), except for the Mini Nutritional Assessment (MNA), which was significantly higher in the leucine group compared to the control group (*p* = 0.041), indicating a slight better nutritional status.

**Figure 1:**
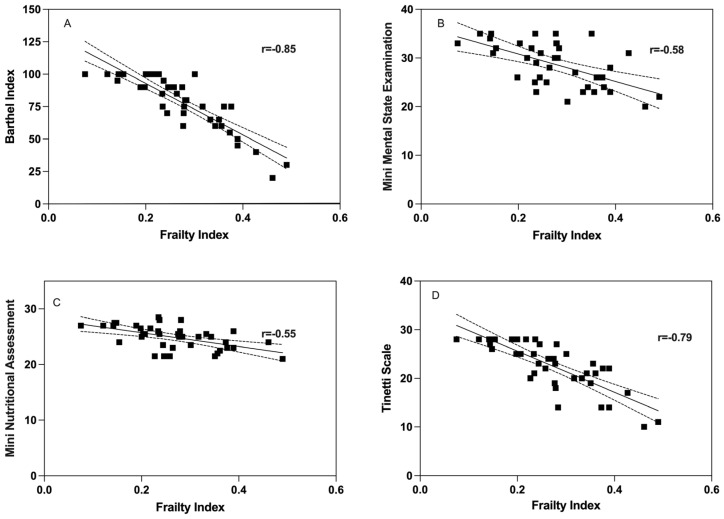
Correlation between Frailty Index at baseline and psycho-geriatric assessments ((**A**) Barthel Index, (**B**) Mini Mental State Examination, (**C**) Mini Nutritional Assessment, (**D**) Tinetti Balance and Gait Evaluation).

The correlation between the Frailty Index at baseline and psychogeriatric assessments was assessed. There was a significant inverse correlation between the FI and the Barthel index (Rho = −0.85, *p* < 0.001; Spearman’s test) (Figure 1A). Cognitive function (MMSE) showed a significant inverse correlation with FI (Rho = −0.58, *p* < 0.001; Pearson’s test) (Figure 1B). There was a significant inverse correlation between the FI and the Mini Nutritional Assessment (MNA) (Rho = −0.55, *p* = 0.002; Pearson’s test) (Figure 1C). In addition, the Tinetti scale showed a significant inverse correlation with the FI (Rho = −0.79, *p* < 0.001; Spearman’s test) (Figure 1D).

**Figure 2:**
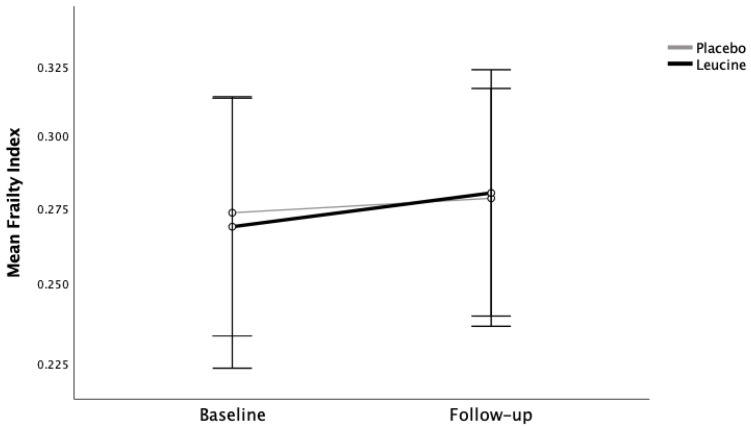
Frailty index levels at baseline and follow-up for the placebo and intervention groups.

There was no statistical difference in FI levels at baseline (placebo group FI 0.27 ± 0.08 and leucine group FI 0.27 ± 0.10) or at 13 week follow-up (placebo group FI 0.28 ± 0.10 and leucine group FI 0.28 ± 0.09). Mixed design analysis of variance showed that there was no significant interaction (*p* = 0.631) of time with treatment group for FI. At 12 week follow-up, the placebo group had similar FI levels as the intervention group (*p* = 0.960) (Figure 2). There was no significant difference between the leucine group and the placebo group in the mean difference in FI between baseline and follow-up (*p* = 0.316; Cohen’s d: 0.04).

There was a significant inverse correlation between the FI and handgrip strength (Rho = −0.383, *p* = 0.014; Spearman’s test) and gait speed (Rho = −0.612, *p* < 0.001; Spearman’s test) both at baseline and at follow-up handgrip strength (Rho = −0.426, *p* = 0.006; Spearman’s test) and gait speed (Rho = −0.708, *p* < 0.001; Spearman’s test). There was no correlation between muscle mass index and FI at baseline (Rho = 0.017, *p* < 0.922; Spearman’s test) nor at follow-up (Rho = −0.182, *p* < 0.295; Spearman’s test).

## 4. Discussion

There have been RCT examining the effect of nutritional intervention, and this trail was original in terms of observation of leucine administration in older institutionalized individuals using the FI tool. Confirming the relationship between psycho-geriatric assessment and frailty in community dwelling individuals found in several studies, we observed and inverse relationship between geriatric rating scales and the frailty index also in institutionalized older individuals, who represented an important group of older individuals who have seldom been analyzed in terms of RCT directed towards improving frailty. This result supports the notion that FI is related to a failure model of ageing systems [4,5,6].

Our data from a RCT with a small sample size showed that the severity of frailty measured by the Frailty Index in nursing home residents was not significantly reduced by a 13 week use of l-leucine supplementation. Nevertheless, in this RCT, previous results [25] reported a significant improvement in some criteria of sarcopenia in the leucine group versus the placebo group, such as functional performance measured by walking time, as well as lean mass index, suggesting that the reduced sample size is not necessarily the cause of the non-significant changes in frailty index after leucine supplementation. The lack of significant differences in FI cannot be attributed to differences in functional, psychological, and nutritional status at baseline. The only difference between the two groups at baseline was in nutritional status, which was slightly better in the leucine group, although both groups had an adequate nutritional status. We cannot rule out that leucine supplementation could have afforded some beneficial effect in FI in individuals with a worse nutritional status compared with those enrolled in the present RCT.

These results are consistent with those described by other authors, who showed a significant negative association between BCAAs and frailty scores, suggesting a potentially protective effect of circulating BCAAs on peripheral leukocyte telomere length (LTL) [7].

It is well known that BCAAs are essential in the maintenance of muscle content and anabolic effects because they improve protein synthesis and reduce the rate of protein degradation [8,9,10]. BCAA ingestion stimulates the activation of mTORC1 signaling pathways that regulate the translational activity of MPS [10]. The effects of BCAA are due to their effects upon signal transduction signal such as MTOR, which in turn depends upon the background nutritional composition. The effects of BCAA are influenced by imbalances with other amino acids, particularly tryptophan. The effects of BCAA on protein synthesis and muscle mass depend upon sufficient dietary protein and essential amino acid intake [10,11].

Why improvements occur in sarcopenia and not in frailty can be attributed to different reasons. Both sarcopenia and frailty are geriatric conditions that induce a loss of functionality and independence [12]. There is an overlap between the two conditions, particularly with regard to the physical aspects of the frailty phenotype: low grip strength, gait speed, and muscle mass, which predisposes older adults to a wide range of negative health-related incidents. Sarcopenia is therefore a muscle-based concept, related to a loss of muscle mass and function, whereas frailty can be defined as multi-system impairment state associated with increased vulnerability to stressors, including a loss of muscle mass [13].

Sarcopenia may be both the biological basis of physical frailty (FP) and the pathophysiological origin based on which the negative health outcomes of FP develop [14]. One of the objectives for controlling the progression of frailty is therefore to improve skeletal muscle mass loss by developing interventions to prevent or delay its progression [13,29].

Although there are a number of tools for measuring frailty [3,28,30,31,32,33], two of the most commonly used are the frailty phenotype proposed by Fried and colleagues [4] and the FI proposed by Mitnitski and coworkers [3]. The underlying frailty within them is not the same. The frailty phenotype presents a clinical manifestation based on five predefined signs/symptoms related to physical evidence. Meanwhile, the FI consider frailty as a heterogeneous state captured during the aging process [2,15]. This requires a comprehensive assessment of the person for computing the FI, which may consequently resemble a surrogate of biological age [15]. Because of the differences in these frailty measurement tools, frailty may have changed in the intervention group if the frailty phenotype had been used.

Because frail persons, and especially institutionalized residents, are at a high risk of undernutrition and physical inactivity, identifying prompt nutrition and exercise interventions to reserve and maintain muscle mass in frail older adults is crucial [16].

Several studies have shown that protein supplementation alone attenuates the decline in measurements of muscle mass, strength, and function in pre-frail and frail older people measured by Fried’s physical phenotype of frailty [20,21]. For this reason, we cannot exclude that analysis of frailty with different tools more focused on physical deficits could have led to different results.

A combination of exercise and protein supplementation seems to have greater impact in attenuating loss of muscle mass, strength, and function in frail older people, as shown by clinical trials that provided a significant effect by using exercise programs with nutritional interventions compared with the effect afforded by nutritional supplementation alone [11,22,34,35]. However, a recent trial by Roschel and co-workers [36] that evaluated the leucine supplementation (7.5 g/day) vs. placebo (alanine) in a different population, namely, 44 community-dwelling older individuals, failed to show any significant change in muscle function and lean mass after 16 weeks of resistance training combined with leucine or placebo supplementation. Amasene et al. [37] evaluated the effects of a resistance training program with post-exercise leucine-enriched protein supplementation on sarcopenia and frailty status in post-hospitalized older adults. The program lasted for 12 weeks, and physical function was assessed by the handgrip strength and the Short Physical Performance Battery, with the results failing to report statistically significant differences between groups, which in turn partially supports the results of our study (leucine alone without a supervises exercise program).

A long-term (48 week) prospective randomized controlled trial in older people with type 2 diabetes evaluated the effect of a resistance exercise program plus supplementation with 6 g of a leucine-rich amino acid compared to physical exercise alone. No additive effect of leucine-rich amino acid supplementation on strength or muscle mass was observed [38].

Leucine-enriched protein (not leucine alone) supplementation appears to show benefits when co-administered with vitamin D, e.g., improvements in muscle mass and lower-extremity function among sarcopenic older adults [24,26].

Moreover, a systematic review identified that protein supplementation plus multicomponent exercise training (MET) had significant effects on diminishing frailty, whereas protein supplementation plus resistance exercise training RET exerted additional effects on muscle mass gain [39]. In addition, the duration of the intervention, classifying the periods as short (12 weeks), medium (12 to 24 weeks), or long (24 weeks), seems to have some influence on the effectiveness of the treatment [39], and for these reasons we cannot exclude the fact that leucine supplementation may benefit frailty index scores after longer period of treatments. The RCT presents several limitations. As already published by our group for the primary outcome of this RCT [23] in order to achieve 90% power with a two-sided 5% level of significance, as well as to detect a minimum difference of one sarcopenia parameter between the placebo and leucine groups in the design of the primary outcome of the study, we calculated that a sample size of a total of 44 patients would enter this two-treatment crossover [23,34]. Due to the fact that the effect of leucine supplementation in frailty index was a secondary outcome, we lost three individuals (N = 41) compared to data we have already published about the effect of leucine supplementation in sarcopenia (skeletal and respiratory muscle sarcopenia) [23]. In this sense, the present results should be considered a proof-of-concept study. In addition, the population sample enrolled in the present study was characterized by low physical activity and most had a fairly sedentary lifestyle; thus, we cannot exclude the same dosage and duration of treatment could add beneficial effects in frailty in a more physically active group of older individuals. Even with the limitation of the reduced sample size, the goal of this secondary analysis of a RCT represents an early proof of concept study, as these studies typically involving a small number of subjects, aiming provide evidence that a drug is likely to be successful or not in later stages of drug development [35]. Although often not published, such studies allow drug developers to make suggestions about proceeding with larger, more expensive studies. Even though we were not able to see any significant or detrimental effect, it is important to let the scientific community let know these results in order to give some insight about the design of future RCTs using leucine supplementation in frailty such as testing higher doses of leucine, longer duration of treatment, or different clinical profiles of participants.

## 5. Conclusions

Leucine supplementation did not significantly change the frailty index levels in nursing home residents. Further studies are recommended to explore the association with other diet component or supplementation. Since frailty index represents a multidimensional construct of frailty, it can be plausible that supplementation with leucine can afford some effect in physical phenotype of frailty syndrome.

## Figures and Tables

**Table 1:** Descriptive characteristics of the study sample.

Category	Placebo	Leucine	*p*-Value
N	23	18	
Age (mean ± SD)	78.3 ± 8.7	78.1 ± 9.6	0.935
N (%) females	14 (60.9)	13 (72.2)	0.520
Mini Mental State Examination (MMSE)	27.8 ± 5.0	29.5 ± 3.9	0.255
Mini Nutritional Assessment (MNA)	25.4 ± 2.0	24.1 ± 2.0	0.041 *
Ability to perform daily activities (Barthel index)	78.0 ± 21.5	79.7 ± 21.3	0.805
Tinetti mobility test (TMT)	23.1 ± 4.8	21.9 ± 5.2	0.472
Comorbidities (Charlson Index)	5.34 ± 2.0	5.0 ± 1.7	0.576
Baseline Frailty Group N (%)			
0–0.2	5 (21.7%)	4 (22.2%)	0.721
0.2–0.3	9 (39.1%)	9 (50.0%)	
0.3+	9 (39.1%)	5 (27.8%)	
Follow-up Frailty Group N (%)			
0–0.2	5 (21.7%)	3 (16.7%)	0.808
0.2–0.3	8 (34.8%)	9 (44.4%)	
0.3+	10 (43.5%)	7 (38.9%)	
Frailty Index Difference (mean ± SD)	0.005 ± 0.45	0.01 ± 0.38	0.316

* The values are represented as means ± SEM; * statistically significant greater reduction in the frailty index compared to the other two frailty groups (*p* < 0.05).

## Data Availability

Data will be made available on request to the corresponding author.
